# Medial Patellofemoral Ligament Reconstruction During Primary Total Knee Arthroplasty in Patients With Pre-existing Patellar Instability: Surgical Technique

**DOI:** 10.1016/j.artd.2026.102107

**Published:** 2026-08-17

**Authors:** Blake E. Delgadillo, Rex Lutz, Bradford Tucker, Zachary Post, Danielle Ponzio, Alvin Ong

**Affiliations:** a Department of Orthopaedic Surgery, Jefferson Health, Stratford, NJ, USA; b Department of Orthopaedic Surgery, Rothman Orthopaedic Institute, Egg Harbor Township, NJ, USA

**Keywords:** Total knee arthroplasty, Medial patellofemoral ligament reconstruction, Patellar instability, Surgical technique, Extensor mechanism

## Abstract

The medial patellofemoral ligament (MPFL) is vital for patellar stability and tracking within the trochlear groove in early flexion. Although MPFL reconstruction is a well-established treatment for patellar instability in native knees, its role during primary total knee arthroplasty (TKA) remains incompletely defined. This manuscript describes a technique for concurrent MPFL reconstruction during primary TKA in select group of patients with a documented history of chronic patellar instability and persistent intraoperative maltracking despite appropriate component positioning and soft tissue balancing. Key surgical steps include individualized patellar fixation based on bone stock and resurfacing status, isometric femoral tunnel placement, graft tensioning, and selective lateral retinacular lengthening. The described technique provides a reproducible option for addressing persistent patellar instability during primary TKA in highly selected patients with longstanding instability and appropriate implant positioning. Given the risks of arthrofibrosis and the limited available evidence, the procedure should be reserved for very select cases.

## Introduction

The medial patellofemoral ligament (MPFL) is critical for patellar stability and tracking within the trochlear groove, especially early in flexion [1]. When the patella laterally dislocates, there is nearly always injury to the MPFL, which then increases an individual's future risk of patellar instability and dislocation [2]. It is well understood that suboptimal patellofemoral alignment is associated with poor outcomes after total knee arthroplasty (TKA) [3]. Although patellofemoral alignment in TKA is typically achieved through proper positioning of the femoral and tibial components, chronic patella instability may require realignment of the extensor mechanism after all other surgical techniques have been exhausted [4]. Although rare, with an incidence between 0.15 and 0.5%, patellar dislocation following TKA carries significant implications [5]. While MPFL reconstruction is a well-established treatment for patellar instability in native knees, MPFL reconstruction during or after primary TKA is less extensively studied. MPFL reconstruction in native knees has many well documented techniques; however, there is a lack of techniques involving the nuances of this procedure in TKA.

Case reports and case series have demonstrated that MPFL reconstruction may effectively treat patellar maltracking and recurrent dislocation after TKA when bony alignment and component positioning are adequate [[5], [6], [7]]. MPFL reconstruction using autograft or allograft has shown encouraging short-term outcomes, including decreased recurrence of instability, decreased patellar tilt, and increased patient satisfaction [[5], [6], [7], [8]]. Based on the current literature, MPFL reconstruction may be an effective treatment for patellar instability following TKA in patients without bony malalignment or component malposition.

Data regarding concurrent MPFL reconstruction during primary TKA is limited. The results from a small retrospective cohort study found that MPFL reconstruction during primary TKA appears to decrease patellar tilt, restore patellar stability, and improve long-term outcomes secondary to minimizing wear [9]. Additionally, 1 case report described concurrent MPFL reconstruction during primary TKA for a patient with preoperative valgus deformity, and the authors found that the patient had improved range of motion, satisfaction, and functional scores without any additional patellar dislocations [10].

However, concurrent MPFL reconstruction during primary TKA should be considered exceptionally uncommon and reserved only for patients with longstanding patellar instability with persistent patellar maltracking despite optimal implant positioning and soft tissue balancing. Current data are limited to case reports, case series, and small retrospective cohorts with wide variations and minimally described technique. Currently, no standardized intraoperative approach exists regarding this technique. Therefore, the goal of this novel manuscript is to describe a reproducible technique for MPFL reconstruction for patellar instability during primary TKA, highlighting the nuances in the technique that are present during TKA.

## Surgical technique

### Compliance

After obtaining institutional review board approval (#2025-1323), this manuscript was conducted in a Health Insurance Portability and Accountability Act compliant fashion.

### Indications and contraindications

Indications include a history of documented recurrent patellar instability, chronic lateral patellar subluxation/dislocation, valgus deformity with chronic instability, failure of native soft tissue restraints, no evidence of component malposition, no rotational malalignment, and no major coronal malalignment. It should be noted that this technique is not intended for routine management of mild intraoperative maltracking or subtle patellar tilt encountered during primary TKA.

Contraindications include component malpositioning, absence of patellar instability, inadequate bone stock, and soft tissues not amenable to repair.

### Patient positioning and preparation

A pneumatic tourniquet is applied and can be inflated at the discretion of the operating surgeon. If a tourniquet is utilized, an Esmarch should be used to exsanguinate the extremity. The knee is prepped and draped in a sterile fashion. Note: the tourniquet should be deflated prior to assessing patellar tracking.

### Total knee arthroplasty

Next, the TKA is performed through a midline anterior skin incision and medial parapatellar or mid vastus approach. The surgeon may choose to resurface the patella or not, and this will impact future surgical steps, as described later. While trialing before final implants, the surgeon should assess patellar tracking through full range of motion, patellar bone stock, lateral retinacular tightness, and MPFL laxity. Towel clamps may be used to approximate the arthrotomy. If the patella remains unstable in a patient with longstanding patellar instability despite optimal implant positioning and absence of malalignment, MPFL reconstruction with or without lateral retinacular lengthening may be considered (Table 1). If the patella reliably tracks centrally and soft tissues are deemed balanced and adequate, an MPFL reconstruction may not be necessary. Although the decision for MPFL reconstruction is made in the preoperative period after extensive history, clinical, and radiographic evaluation, the final decision should be confirmed intraoperatively with the aforementioned parameters.

**Table 1:** Pearls, pitfalls, and key decisions encountered throughout concurrent TKA and MPFL reconstruction.

Step	Pearls	Pitfalls	Key decision
Patient positioning and preparation	Use wide sterile field for intraoperative flexibility	Inadequate exposure may limit ability to address key steps	Tourniquet vs no tourniquet
Total knee arthroplasty	Patellar tracking should be assessed with trial implants	Overlooking subtle patellar maltracking may lead to postoperative patellar instability	Patellar resurfacing vs no resurfacing
Graft measurement and preparation	Prepare graft slightly longer than anticipated to allow for proper tensioning	Short graft risks inadequate fixation or overtightening	Allograft type
Patellar fixation	Use parallel 2.4 mm guide pins with 1 cm lateral bone bridge	Prefer soft tissue or quadriceps tendon fixation in small, osteopenic, or resurfaced patellae to preserve bone stock and minimize fracture risk	Soft tissue fixation vs transosseous fixation based on patellar bone stock and resurfacing status
Femoral fixation and isometry	Graft should slightly loosen in flexion to avoid overtightening	Incorrect tunnel positioning and resulting disruption of femoral box	Interference screw vs cortical button fixation
Lateral retinacular lengthening or Z-lengthening	Perform only if needed after reassessment of patellar tracking post-MPFL reconstruction	Overlengthening laterally may risk destabilizing the patella or lead to avascular necrosis of the patella	No lengthening vs lateral retinacular lengthening or Z-lengthening
Postoperative management	Weight bear as tolerated in a hinged knee brace locked in extension or knee immobilizer for 6 weeks postoperatively Thereafter, aggressive physical therapy to avoid stiffness	Aggressive and premature range of motion may compromise soft tissue repairs	Weight bearing status and range of motion allowance

### Graft measurement and preparation

**Figure 1:**
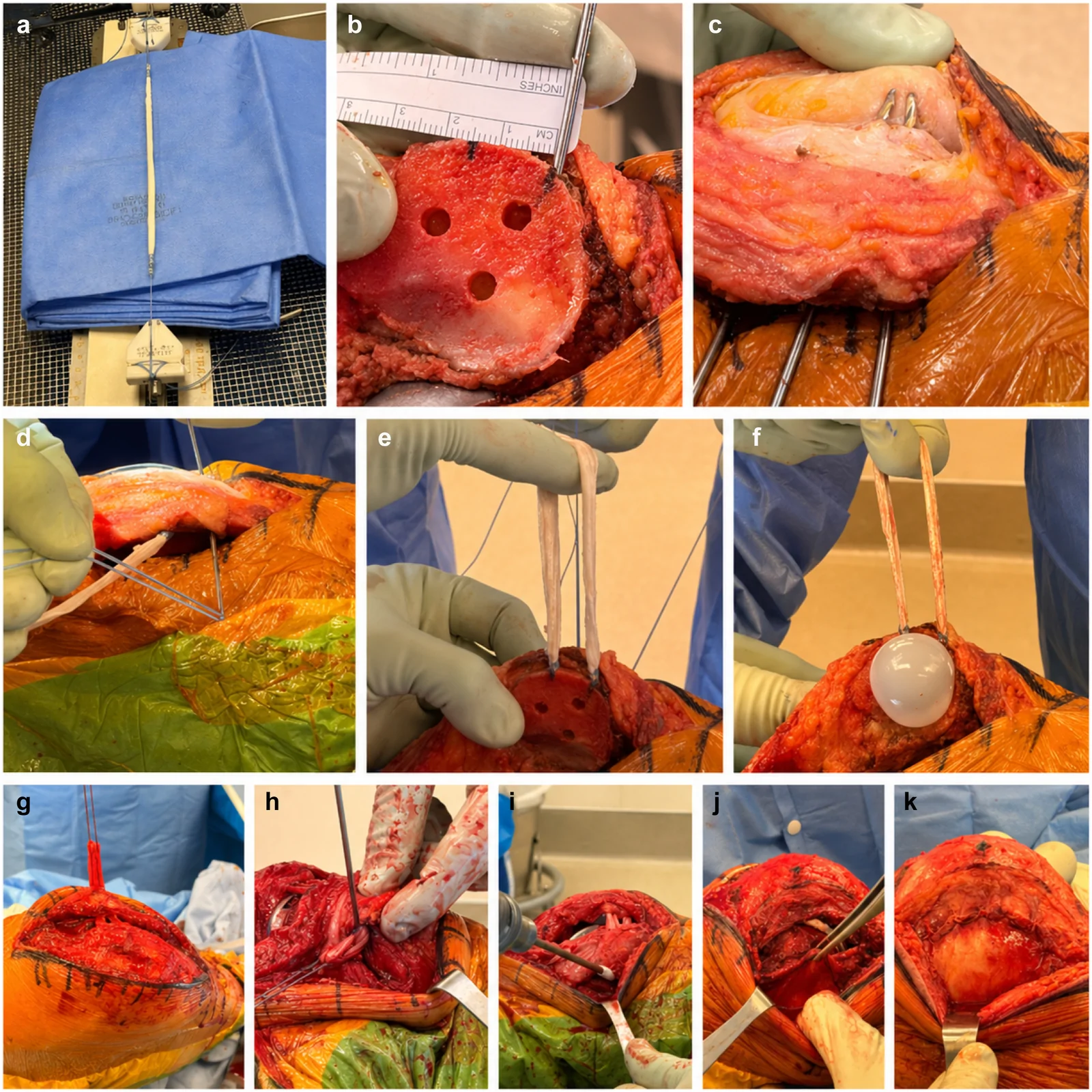
Surgical Technique Pictures. (a) Preparation of graft with a whipstitch using #2 FiberWire (Arthrex, Naples, FL, USA). (b) Patella tunnels are marked 15 mm apart and 2.4 mm pins are placed and over reamed with a 4 mm reamer to a depth of 10 mm. (c) The pins should converge to the lateral bone bridge with care to avoid the predrilled patella button holes. (d and e) The graft ends are passed from medial to lateral and tied over the lateral bone bridge (alternative soft tissue or quadriceps tendon fixation may be preferred in patients with inadequate patellar bone stock). (f) The patella button is fixed to the bone with either pressfit or cement fixation. (g and h) The graft is passed medially between layers 2 and 3 and the medial femoral point of isometry is tested throughout the range of motion. Care is taken to avoid the femoral box during femoral pin placement and it is overreamed. (i) A biocomposite interference screw is placed. If inadequate purchase is encountered, a lateral button can be used. (j) Patella tracking and lateral tightness is assessed. A lateral arthrotomy and lengthening can be performed if there is a lateral retinacular contracture. (k) Final closure of the lateral lengthening in a lengthened position.

After measuring from the intended patellar fixation points to the proposed femoral insertion between the medial epicondyle and adductor tubercle with the knee flexed to roughly 30°, the surgeon should measure the intended length of graft needed for MPFL reconstruction (Table 1; Fig. 1). It is wise to prepare a slightly longer graft than intended to allow for fixation/tensioning. Allograft choices typically include tibialis anterior, posterior tibialis, or semitendinosus tendons. The graft may be doubled to ensure roughly 7-8 mm of diameter is achieved. Both ends of fixation on the graft should be whipstitched with #2 FiberWire (Arthrex; Naples, FL, USA). The grafts may then be soaked in vancomycin ± gentamicin (or saline) per institutional protocol (Table 1).

### Patellar fixation

The proximal-medial starting points may then be marked in the location where 2 tunnels starting 15 mm apart will be (Table 1; Fig. 1). Next, 2 2.4 mm guide pins may be inserted from proximal-medial patella directed to the anterolateral patella leaving a roughly 1 cm bone bridge laterally if bone stock allows. The 2 2.4 mm pins may then be overreamed with a 4 mm reamer to roughly 10 mm depth, although this may be adjusted depending on patellar thickness and width. Next, the graft ends are passed into the tunnels and tied over the lateral bone bridge. Additionally, the grafts may be secured with anchors if there is adequate bone, such as in an unresurfaced patella. If the patella is small, osteopenic, thin, or resurfaced, transosseous fixation should be avoided due to risk of iatrogenic patellar fracture. In these situations, the authors prefer soft tissue fixation or quadriceps tendon-based fixation techniques to preserve patellar bone stock and reduce fracture risk. Transosseous fixation is reserved for patients with adequate patellar bone stock and sufficient patellar thickness to safely accommodate tunnels.

### Femoral fixation and isometry

Next, a small fascial incision over the medial epicondyle. From this region, develop an extra-articular soft tissue tunnel over the joint capsule between layers 2 and 3 toward the main arthrotomy. A passing suture should then be used for later shuttling of the MPFL graft. The MPFL footprint is then identified on the proximal–posterior aspect of the medial epicondyle. Of note, Schottle’s point is not routinely radiographically assessed in this setting given the potential for an altered knee joint line, anatomy, and kinematics. Therefore, the femoral fixation point is identified by palpation and isometric testing. A guide pin is then placed in the footprint and aimed slightly proximal–anterior to avoid entering the box of the femoral component in posterior stabilized TKAs. With the graft fixed on the patella and passed through the soft tissue tunnel, loop the graft over the guide pin, and then reduce the patella in the trochlear groove of the femoral component to test the isometry. With the knee at 30° flexion, the graft can be marked at the level of the pin. Then, the knee should be taken through a full range of motion to evaluate isometry (Table 1; Fig. 1). The goal is for slight loosening of the graft in flexion to avoid overconstraint of the patellofemoral joint. The pin position should be adjusted until appropriate isometry is obtained. Then, the femoral socket should then be overreamed to the diameter of the graft, which is roughly 7-8 mm depending on the length of the graft and size of the interference screw. The graft can then be passed through the soft tissue tunnel into the femoral socket. With the knee at 30° flexion and an anatomically reduced patella, the graft should be appropriately tensioned and secured with an interference screw in patients with good bone stock. If the patient has poor bone stock, the fixation should be augmented with a cortical button or by tying the passing sutures over a button on the lateral cortex. After femoral fixation, the knee should then be taken through full range of motion while assessing patellar tracking and medial–lateral patellar stability. If the patella is too tight laterally or laterally tilted, then perform a lateral retinacular lengthening, not a release.

### Lateral retinacular lengthening or Z-lengthening

The lateral side must be assessed after the medial reconstruction. If the lateral retinacular tension remains excessive and the patella is laterally tilted, then a lateral retinacular lengthening or Z-lengthening should be done (Table 1; Fig. 1). Z-Lengthening involves peeling layers 1-2 off the capsule, cutting the capsule roughly 2 cm lateral to the patellar border, and reapproximating the tissues in a lengthening position to reduce lateral tethering. Additionally, if there is a vastus lateralis contracture or lateral patellar tethering by the vastus lateralis, it can be released proximally off the intermuscular septum prior to repairing the Z-lengthening at the appropriate balance.

### Closure and dressing

The surgical site should then be copiously irrigated. Hemostasis should be confirmed prior to closure. Next, the arthrotomy and fascial incisions should then be closed with absorbable sutures, such as 0 Vicryl (Ethicon; Somerville, NJ, USA) (Table 1; Fig. 1). The subcuticular/subcutaneous layers can then be closed with 2-0 Vicryl or Monocryl (Ethicon; Cornelia, GA, USA). Skin staples or a running dermal closure with 2-octyl cyanoacrylate may be used. A sterile dressing can then be applied per surgeon preference.

### Postoperative management

After applying a soft dressing and ace bandage, the patient should be placed in a knee immobilizer or hinged knee brace locked in extension for the immediate postoperative period. The operative team recommends making the patient weight bearing as tolerated with a rolling walker in a hinged knee brace locked in extension or knee immobilizer for 6 weeks postoperatively. At that point, the bracing may be discontinued and full weight bearing may be resumed with discontinuation of assistive devices once safe and cleared to do so. There should be aggressive physical therapy done to avoid stiffness. If a manipulation under anestehsia must be done, it is recommended to avoid doing so within the first 6 weeks postoperatively.

## Discussion

Patellar instability after TKA is a challenging complication, specifically in patients with risk factors, such as trochlear dysplasia, valgus deformity, patella alta, or chronic subluxation [6,7,11]. In both native and prosthetic knees, it is well-established the MPFL is vital for optimal patellar tracking in the trochlear groove, especially early in flexion [1]. With regard to patellar tracking in TKA, patellofemoral alignment is usually achieved with proper positioning of the prosthetic components, but persistent maltracking despite adequate positioning may necessitate extensor mechanism realignment [4].

To the best of our knowledge, the present manuscript is among the first detailed descriptions of a reproducible surgical technique of concurrent MPFL reconstruction with primary TKA. Technical considerations are paramount to successful outcomes and avoiding complications. For example, to avoid overconstraint, flexion contracture, and graft failure, it is vital to place anatomic femoral tunnels, individualize patellar fixation (transosseous tunnels, suture anchors, or soft tissue reconstruction) depending on various patient factors (patellar thickness, patellar bone stock, and patellar resurfacing status), and carefully assess graft isometry and tension [6,7,12]. Schottle’s point is intentionally not used for femoral tunnel placement in this technique as the altered joint line and kinematics following TKA may be associated with an unreliable radiographic assessment. Furthermore, the femoral component may inhibit radiographic evaluation. Therefore, the femoral fixation point should be palpation-guided as excessive tension or nonanatomic tunnel positioning may lead to persistent pain, recurrent instability, and suboptimal outcomes [7,10,12].

In a small illustrative institutional experience of 5 patients, no recurrent patellar dislocations were observed at short-term follow-up. However, postoperative stiffness requiring MUA occurred in 3 patients, which highlights the substantial risk of arthrofibrosis associated with this procedure. This stiffness may be related to increased soft tissue dissection, postoperative scarring, or prolonged immobilization. Furthermore, this highlights the altered biomechanics that these patients experience preoperatively and thus continue to experience in the postoperative state, remembering that the primary goal is pain relief. Surgeons and patients alike should be aware of the increased risk of arthrofibrosis following this procedure.

The present manuscript is not without limitations. Although the objective was to describe a surgical technique, it lacks prospective clinical and patient-reported outcomes with long-term outcomes. Additionally, the existing literature supporting this approach is limited to minimal case reports. Therefore, clinical interpretation must be taken with caution.

The described technique of MPFL reconstruction during primary TKA may be a feasible and effective strategy for restoring patellar stability in patients undergoing TKA with a history of patellar instability. All patients had a documented history of chronic patellar instability and intraoperative confirmation of persistent patellar maltracking despite optimal component positioning, which further supported MPFL reconstruction. This approach is consistent with the current consensus that MPFL reconstruction is most effective for patellar instability in TKA in the absence of component malrotation, assists in decreasing rates of recurrent instability, and helps improve patient-reported outcomes in both native and prosthetic knees [6,7,10,13,14].

## Summary

The described technique provides a reproducible option for addressing persistent patellar instability during primary TKA in highly selected patients with longstanding instability and appropriate implant positioning. Given the risks of arthrofibrosis and the limited available evidence, the procedure should be reserved for well-indicated cases and further investigation is warranted.

## Conflicts of interest

The authors declare the following financial interests/personal relationships which may be considered as potential competing interests: Alvin Ong reports receiving consulting fees from Smith & Nephew and Stryker, research support from Zimmer; receiving speakers bureau honoraria and consulting fees from Smith & Nephew and Stryker, as well as research support and publisher-related financial support from Smith & Nephew; and serving on the editorial or governing boards of The Journal of Arthroplasty and Journal of Orthopaedic Surgery and Research. Zachary Post reports receiving speaker’s bureau honoraria from Ortho Development and consulting fees from Ortho Development and DePuy. Rex Lutz reports serving as Chair of the AAOS Resident Assembly Executive Committee. The remaining authors declare that they have no known competing financial interests or personal relationships that could have appeared to influence the work reported in this paper.

For full disclosure statements, refer to https://doi.org/10.1016/j.artd.2026.102107.

## CRediT authorship contribution statement

**Blake E. Delgadillo:** Writing – review & editing, Writing – original draft, Visualization, Validation, Software, Resources, Project administration, Methodology, Investigation, Funding acquisition, Formal analysis, Data curation, Conceptualization. **Rex Lutz:** Writing – review & editing, Visualization, Validation, Supervision, Software, Resources, Project administration, Methodology, Investigation, Funding acquisition, Formal analysis, Data curation, Conceptualization. **Bradford Tucker:** Writing – review & editing, Visualization, Validation, Supervision, Project administration, Methodology, Investigation, Conceptualization. **Zachary Post:** Writing – review & editing, Supervision, Project administration. **Danielle Ponzio:** Writing – review & editing, Supervision, Project administration. **Alvin Ong:** Writing – review & editing, Supervision, Project administration.

## Funding

The Thomas Jefferson Universtiy Article Processing Charge fund was used to fund publication fees.

## Appendix A Supplementary data

Conflict of Interest Statement for Ong

Conflict of Interest Statement for Delgadillo

Conflict of Interest Statement for Ponzio

Conflict of Interest Statement for Lutz

Conflict of Interest Statement for Post

Conflict of Interest Statement Tucker
