# Supplementary material for: Medial Patellofemoral Ligament Reconstruction During Primary Total Knee Arthroplasty in Patients With Pre-existing Patellar Instability: Surgical Technique
Source: Arthroplast Today. 2026 Aug 17;41:102107. doi: 10.1016/j.artd.2026.102107 (PMC13506795; doi:10.1016/j.artd.2026.102107)
Supplement: Supplementary file 5 [file mmc5.docx]

# INDIVIDUAL CONFLICT OF INTEREST STATEMENT

***The Journal of Arthroplasty***

(Adopted from the American Academy of Orthopaedic Surgeons disclosure statement)

The following form **must be filled out completely and submitted by each author (example, 6 authors, 6 forms).**

**All items require a response. If there is no relevant disclosure for a given item, enter "*None*.”**

**Preoperative SGLT2 Inhibitor Use is Associated with Increased Risk of Complications following Total Joint Arthroplasty: A Propensity Score-Matched Analysis**

1.	Royalties from a company or supplier (The following conflicts were disclosed)

None

2.	Speakers bureau/paid presentations for a company or supplier (The following conflicts were disclosed)

Ortho development

3A.	Paid employee for a company or supplier (The following conflicts were disclosed)

None

3B.	Paid consultant for a company or supplier (The following conflicts were disclosed)

Ortho development, Depuy

3C.	Unpaid consultants for a company or supplier (The following conflicts were disclosed)

None

4.	Stock or stock options in a company or supplier (The following conflicts were disclosed)

None

5.	Research support from a company or supplier as a Principal Investigator (The following conflicts were disclosed)

None

6.	Other financial or material support from a company or supplier (The following conflicts were disclosed)

None

7.	Royalties, financial or material support from publishers (The following conflicts were disclosed)

None

8.	Medical/Orthopaedic publications editorial/governing board (The following conflicts were disclosed)

None

9.	Board member/committee appointments for a society (The following conflicts were disclosed)

None

**Each author must sign AND print or type his/her name, date and submit a separate form**

In addition, one BLINDED Conflict of Interest form (no author names used) should be submitted per manuscript with all author disclosures.

Zachary Post	Zachary Post	4/30/2026

Author Name (Print or Type)	Author Signature	Date
